# Supplementary material for: Synthesis of glycoconjugates utilizing the regioselectivity of a lytic polysaccharide monooxygenase
Source: Sci Rep. 2020 Aug 6;10:13197. doi: 10.1038/s41598-020-69951-7 (PMC7411024; doi:10.1038/s41598-020-69951-7)
Supplement: Supplementary file 1 — Supplementary Information. [file 41598_2020_69951_MOESM1_ESM.pdf]

Synthesis of Glycoconjugates Utilizing the Regioselectivity of a Lytic Polysaccharide Monooxygenase

Bjørge Westereng, a* Stjepan K. Kračun, b Shaun Leivers, a Magnus Ø. Arntzen, a Finn L. Aachmann, c,* and Vincent G. H. Eijsink, a.

[a] Dr B, Westereng*, Dr Shaun Leivers, Dr M Arntzen, Prof V.G.H., Eijsink; Faculty of Chemistry, Biotechnology and Food Science, NMBU - Norwegian University of Life Sciences, Chr.M.Falsens vei 1, Aas, Norway.

[b] Dr SK Kračun; Department of Plant and Environmental Sciences, University of Copenhagen, Thorvaldsensvej 40, DK-1871 Frederiksberg C, Denmark

[c] Prof F L., Aachmann*; Department of Biotechnology and Food Science, NTNU - Norwegian University of Science and Technology, Sem Sælands vei 6/8, N-7491 Trondheim, Norway.

Supplementary Material

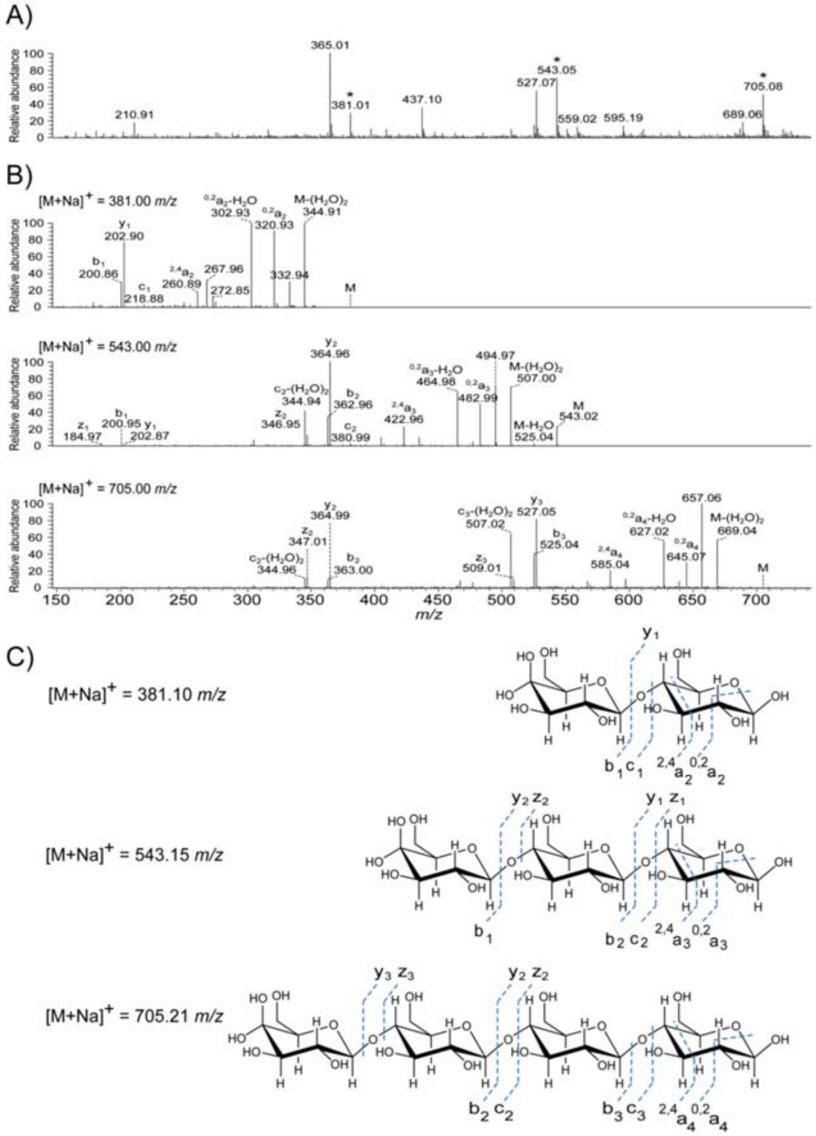

Figure S1. **Direct injection MS analysis of C4-oxidized cello-oligosaccharides.** Oxidized soluble products were analyzed using an LTQ Velos Pro mass spectrometer and direct injection. A) A survey scan of all ions detected in the *m/z*-range 150-750. Peaks annotated with an asterisk are sodium adducts of the hydrated forms of oxidized products and were selected for fragmentation. Note that the keto-sugars are in equilibrium with their gemdiols and that under the conditions used in this study, the keto-form is predominant in MALDI ToF MS analysis (Fig. 1), whereas the gemdiol is predominant in ESI-MS. Peaks corresponding to native cello-oligosaccharides are also visible (*m/z* 365, 527 and 689), which is due to the samples being taken at an early time point in the reaction when there is some release of non-modified sugars from chain ends, while the oxidized oligosaccharides are mostly still bound to the polymer part of the cellulose. B) MS/MS spectra of the three C4-oxidized products show a dominant cleavage of the glycosidic bonds and ring cleavage of the downstream end-sugar. Fragments including the C4-oxidation (a,b,c fragments) tend to lose water as has been described previously [1]. This characteristic double loss of water from the molecular mass is evident for all three products. The MS fragmentation pattern observed here as well as the elution pattern in HPAEC-PAD chromatography are in accordance with previous observations on NMR validated C4-oxidized cello-oligosaccharides [1]. C) The structures of the three C4-oxidized products; the observed fragmentation patterns and the theoretical masses are indicated.

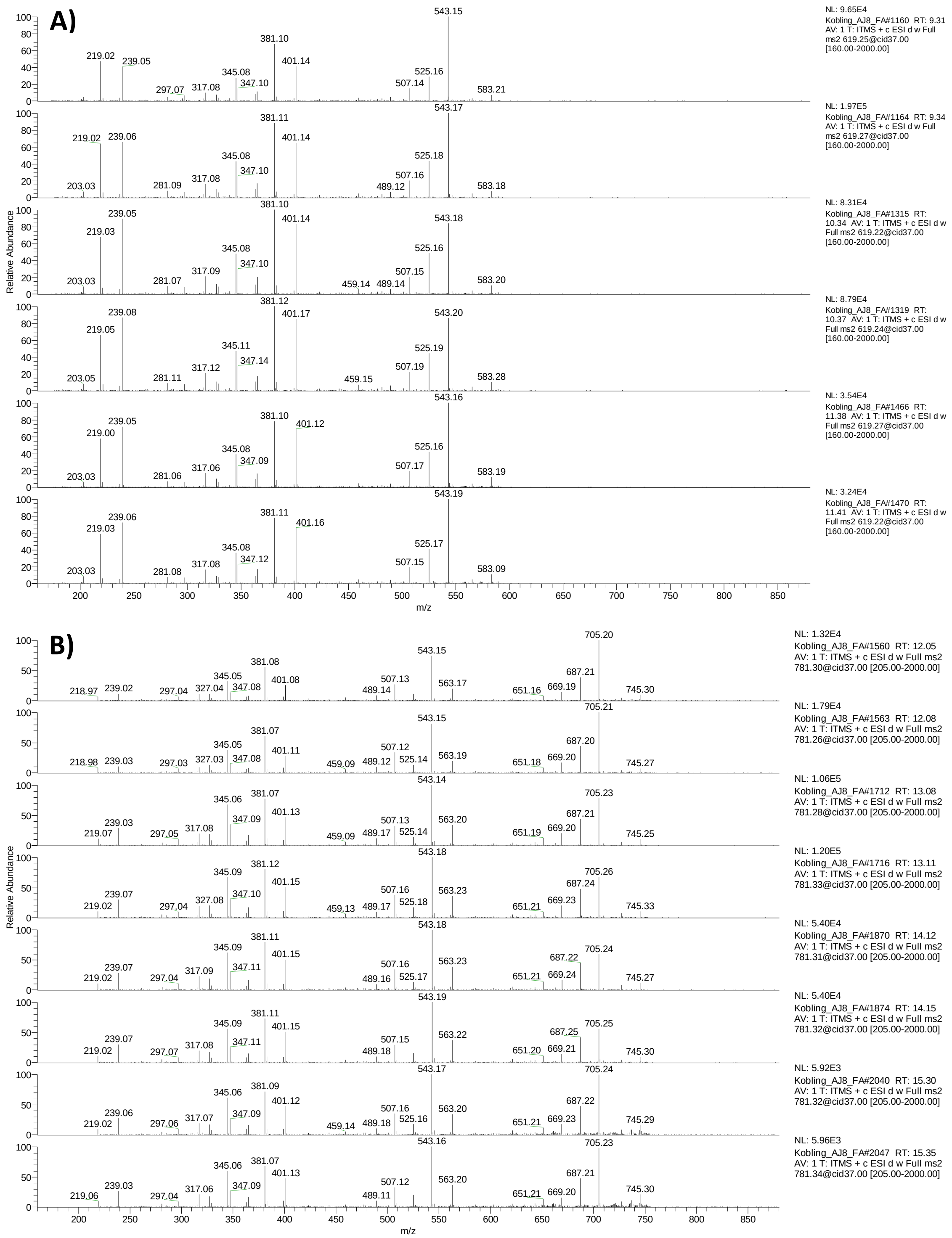
A)
B)
Relative Abundance
m/z
NL: 9.65E4 Kobling_AJ8_FA#1160 RT: 9.31 AV: 1 T: ITMS + c ESI d w Full ms2 619.25@cid37.00 [160.00-2000.00]
NL: 1.97E5 Kobling_AJ8_FA#1164 RT: 9.34 AV: 1 T: ITMS + c ESI d w Full ms2 619.27@cid37.00 [160.00-2000.00]
NL: 8.31E4 Kobling_AJ8_FA#1315 RT: 10.34 AV: 1 T: ITMS + c ESI d w Full ms2 619.22@cid37.00 [160.00-2000.00]
NL: 8.79E4 Kobling_AJ8_FA#1319 RT: 10.37 AV: 1 T: ITMS + c ESI d w Full ms2 619.24@cid37.00 [160.00-2000.00]
NL: 3.54E4 Kobling_AJ8_FA#1466 RT: 11.38 AV: 1 T: ITMS + c ESI d w Full ms2 619.27@cid37.00 [160.00-2000.00]
NL: 3.24E4 Kobling_AJ8_FA#1470 RT: 11.41 AV: 1 T: ITMS + c ESI d w Full ms2 619.22@cid37.00 [160.00-2000.00]
NL: 1.32E4 Kobling_AJ8_FA#1560 RT: 12.05 AV: 1 T: ITMS + c ESI d w Full ms2 781.30@cid37.00 [205.00-2000.00]
NL: 1.79E4 Kobling_AJ8_FA#1563 RT: 12.08 AV: 1 T: ITMS + c ESI d w Full ms2 781.26@cid37.00 [205.00-2000.00]
NL: 1.06E5 Kobling_AJ8_FA#1712 RT: 13.08 AV: 1 T: ITMS + c ESI d w Full ms2 781.28@cid37.00 [205.00-2000.00]
NL: 1.20E5 Kobling_AJ8_FA#1716 RT: 13.11 AV: 1 T: ITMS + c ESI d w Full ms2 781.33@cid37.00 [205.00-2000.00]
NL: 5.40E4 Kobling_AJ8_FA#1870 RT: 14.12 AV: 1 T: ITMS + c ESI d w Full ms2 781.31@cid37.00 [205.00-2000.00]
NL: 5.40E4 Kobling_AJ8_FA#1874 RT: 14.15 AV: 1 T: ITMS + c ESI d w Full ms2 781.32@cid37.00 [205.00-2000.00]
NL: 5.92E3 Kobling_AJ8_FA#2040 RT: 15.30 AV: 1 T: ITMS + c ESI d w Full ms2 781.32@cid37.00 [205.00-2000.00]
NL: 5.96E3 Kobling_AJ8_FA#2047 RT: 15.35 AV: 1 T: ITMS + c ESI d w Full ms2 781.34@cid37.00 [205.00-2000.00]

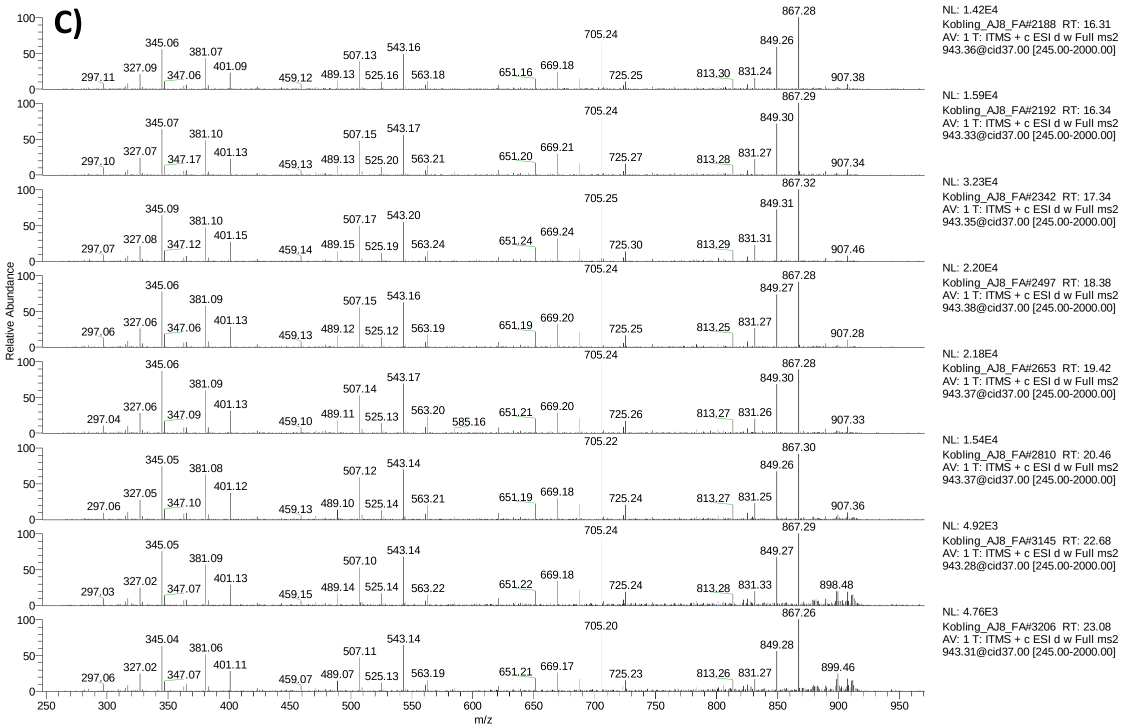

Figure S2**. PGC-MS/MS analysis of the oxime bi-functionalized cello-oligosaccharides displaying fragmentation data on the three peaks in Fig. 6A.** A) Fragments of 619.25 from RT 9.31 to 11.41, B) Fragments of 781.28 from RT 12.05 to 15.35, and C) Fragments of 943.35 from RT 16.31 to 23.08

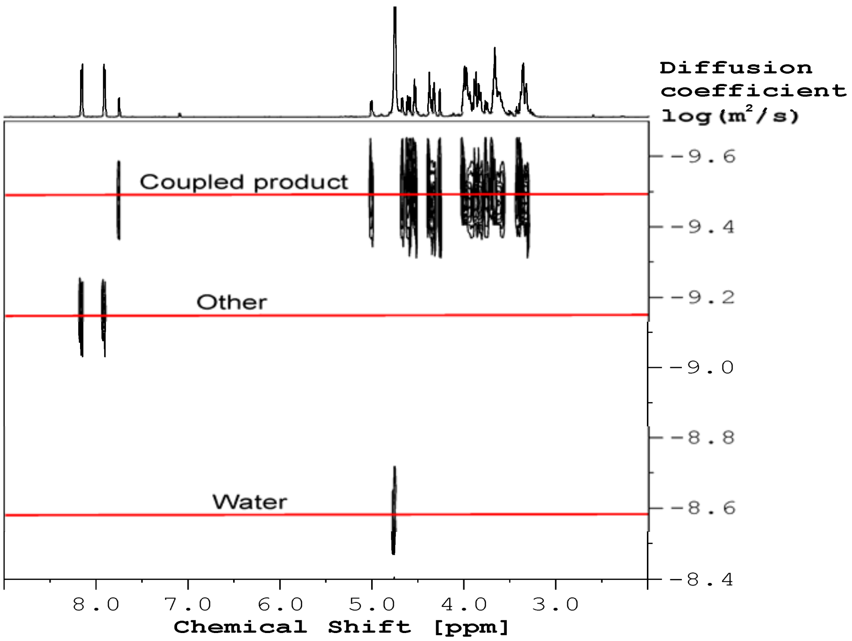

Figure S3**. Diffusion-Ordered Spectroscopy (DOSY) on the aminooxy-linked product (notion coupled product in spectrum)**. DOSY spectrum of the aminooxy-linked product in 99.9 % D_2O recorded at 25°C. Red lines indicate the diffusion of the different molecules in the sample. If the aminooxy-linked products have the same diffusion coefficient it indicates that the C4-oxidized Glc and the oxime linker are covalently linked, while, if they have a different diffusion coefficient, they are not covalently bonded at all. Thus DOSY provides an indirect qualitative method to validate if a coupling reaction has been achieved and helps to identify signals belonging to the aminooxy-linked product (more specifically the azomethine (-C=N-) group which is formed upon coupling). A complete structural elucidation by NMR (Fig.6) was further performed to obtain direct proof of the structure for the coupled product. The [^1H-^{13}C] HMBC spectrum provided long range bond correlations allowing to connect the monosaccharides for the aminooxy-linked product as well as identification of the carbon chemical shift for a C4-oxidized end at C4 (see figure 6A).

Table S1: The chemical shift values and correlations of the aminooxy-linked product. Chemical shifts were assigned for the main product in 99.9% D_2O at 25 °C. Numbers 1-6 represent ring carbon numbers to which the chemical shift values [^{1}H,^{13}C] are assigned. The water signal (4.75 ppm) was used as chemical shift reference for ^{1}H and ^{13}C was referenced indirectly using the absolute frequency ratio [2].

Position	^{1}H [ppm]	^{13}C [ppm]	COSY correlations - correlated nuclei	HMBC correlations - correlated nuclei
C4ox-1	4.67	107.0	C4ox-2	C4ox-2, C4ox-3, C4ox-5
C4ox-2	3.89	76.7	C4ox-1, C4ox-3	C4ox-1, C4ox-3, C4ox-4
C4ox-3	4.27	73.4	C4ox-2	C4ox-1, C4ox-2, C4ox-4, C4ox-5
C4ox-4	-	159.2		C4ox-2, C4ox-3, C4ox-5, C4ox-6
C4ox-5	5.01	77.1	C4ox-6	C4ox-1, C4ox-3, C4ox-4, C4ox-6
C4ox-6	3.99;3.89	63.5	C4ox-5	C4ox-5, C4ox-4
Glc3-1	4.60	104.9	Glc3-2	Glc3-2, Glc3-3, Glc3-5
Glc3-2	3.39	75.6	Glc3-1, Glc3-3	Glc3-1, Glc3-3, Glc3-4
Glc3-3	3.57	77.2	Glc3-2, Glc3-4	Glc3-1, Glc3-2, Glc3-4, Glc3-5
Glc3-4	3.63	81.0	Glc3-3, Glc3-5	C4ox-1, Glc3-2, Glc3-3, Glc3-5, Glc3-6
Glc3-5	3.64	76.7	Glc3-4, Glc3-6	Glc3-1,Glc3-4, Glc3-3, Glc3-6
Glc3-6	3.95;3.87	62.5	Glc3-5	Glc3-5, Glc3-4
Glc2-1	4.54	105.1	Glc2-2	Glc2-2, Glc2-3, Glc2-5
Glc2-2	3.37	75.7	Glc2-1, Glc2-3	Glc2-1, Glc2-3, Glc2-4
Glc2-3	3.67	76.6	Glc2-2, Glc2-4	Glc2-1, Glc2-2, Glc2-4, Glc2-5
Glc2-4	3.66	81.0	Glc2-3, Glc2-5	Glc3-1, Glc2-2, Glc2-3, Glc2-5, Glc2-6
Glc2-5	3.62	77.5	Glc2-4, Glc2-6	Glc2-1, Glc2-4, Glc2-3, Glc2-6
Glc2-6	3.98;3.84	62.4	Glc2-5	Glc2-5, Glc2-4
Glc1-1	7.76	155.4	Glc1-2	B’, Glc1-2, Glc1-3, Glc1-5
Glc1-2	4.61	71.9	Glc1-1,Glc1-3	Glc1-1, Glc1-3, Glc1-4
Glc1-3	3.99	73.7	Glc1-2,Glc1-4	Glc1-1, Glc1-2, Glc1-4, Glc1-5
Glc1-4	3.95	80.7	Glc1-3,Glc1-5	Glc2-1, Glc1-2,Glc1-3, Glc1-5, Glc1-6
Glc1-5	3.96	73.6	Glc1-4,Glc1-6	Glc1-1, Glc1-3,Glc1-4, Glc1-6
Glc1-6	3.88;3.76	64.6	Glc1-5	Glc1-5
A (NH_2-**CH_2**-CH_2-O-R)	3.34	41.4	B	B
A’ (NH_2-**CH_2**-CH_2-O-R’)	3.29	41.2	B’	B’
B (NH_2-CH_2-**CH_2**-O-R)	4.37	72.9	A	B
B’ (NH_2-CH_2-**CH_2**-O-R’)	4.32;4.01	72.5	A’	Glc1-1, A’

References

1. Agger, J. W. *et al.* Discovery of LPMO activity on hemicelluloses shows the importance of oxidative processes in plant cell wall degradation. *Proc. Natl. Acad. Sci. U. S. A.* **111**, 6287–6292 (2014).
2. Zheng, D. *et al.* Letter to the Editor: ^{1}H, ^{13}C and ^{15}N resonance assignments for methionine sulfoxide reductase B from *Bacillus subtilis*. *J. Biomol. NMR* **27**, 183–184 (2003).
